# Impact of Olfactory Change on Postoperative Body Weight Loss in Patients with Gastric Cancer after Gastrectomy

**DOI:** 10.3390/nu16060851

**Published:** 2024-03-15

**Authors:** Hiromi Matsuo, Ryota Matsui, Koshi Kumagai, Satoshi Ida, Yoko Saino, Aya Fujihara, Kumi Takagi, Yukiko Itami, Misuzu Ishii, Naoki Moriya, Yuna Izumi-Mishima, Kazuhiro Nomura, Yasuo M. Tsutsumi, Souya Nunobe, Rie Tsutsumi, Hiroshi Sakaue

**Affiliations:** 1 Department of Nutrition and Metabolism, Institute of Biomedical Sciences, Tokushima University Graduate School, 3-18-15 Kuramoto, Tokushima 770-8503, Japanyoko.saino@jfcr.or.jp (Y.S.); hsakaue@tokushima-u.ac.jp (H.S.); 2 Department of Nutrition Management, Cancer Institute Hospital of JFCR, 3-8-31 Ariake, Koto-ku, Tokyo 135-0063, Japan; 3 Department of Gastroenterological Surgery, Cancer Institute Hospital of JFCR, 3-8-31 Ariake, Koto-ku, Tokyo 135-0063, Japan; 4 Department of Upper Gastrointestinal Surgery, Kitasato University Hospital, 1-15-1 Kitasato, Minami-ku, Sagamihara-shi 252-0375, Japan; 5 Department of Gastroenterological Surgery, Kumamoto University Hospital, 1-1-1 Honjo, Chuo-ku, Kumamoto-shi 860-8556, Japan; 6 Department of Anesthesiology and Critical Care, Hiroshima University, 1-2-3 Kasumi, Hiroshima 734-8551, Japan

**Keywords:** olfaction, gastric cancer, gastrectomy, body weight loss, smell

## Abstract

Patients undergoing gastrectomy for gastric cancer may experience alterations in olfaction, yet the association between olfactory changes and postoperative weight loss remains uncertain. This study aimed to elucidate the relationship between olfactory changes and postoperative weight loss in patients with gastric cancer. Patients who underwent radical gastrectomy for gastric cancer between February 2022 and August 2022 were included in the study. Those experiencing a higher Visual Analog Scale (VAS) score postoperatively compared to preoperatively were deemed to have undergone olfactory changes. Postoperative weight loss was determined using the 75th percentile as a cutoff value, designating patients surpassing this threshold as experiencing significant weight loss. Multivariate logistic regression analysis was employed to identify risk factors for postoperative weight loss, with statistical significance set at *p* < 0.05. Out of 58 patients, 10 (17.2%) exhibited olfactory changes. The rate of postoperative weight loss at one month was markedly higher in the group with olfactory changes compared to those without (9.6% versus 6.2%, respectively; *p* = 0.002). In addition, the group experiencing olfactory changes demonstrated significantly lower energy intake compared to the group without such changes (1050 kcal versus 1250 kcal, respectively; *p* = 0.029). Logistic regression analysis revealed olfactory changes as an independent risk factor for significant weight loss at one month postoperatively (odds ratio: 7.64, 95% confidence interval: 1.09–71.85, *p* = 0.048). In conclusion, olfactory changes emerged as an independent risk factor for postoperative weight loss at one month in patients with gastric cancer following gastrectomy.

## 1. Introduction

Patients who undergo gastrectomy for gastric cancer often experience postoperative weight loss, which is strongly associated with a decrease in quality of life (QOL) and poor postoperative outcomes. Weight loss occurs due to a decrease in gastric volume and malabsorption in the gastrointestinal tract [1,2]. Previous reports have shown that greater postoperative weight loss is associated with lower rates of adjuvant chemotherapy continuation [3] and worse long-term prognosis [4,5]. In addition, patients with severe post-gastrectomy symptoms or a low postoperative body mass index (BMI) have been reported to have lower postoperative QOL [6]. Therefore, it is important to accurately assess post-gastrectomy symptoms, especially weight loss, to improve prognosis and maintain QOL.

Sensory impairments, such as taste and smell disorders, also greatly contribute to the causes of such weight loss. Although the relationship between smell disorders and gastrectomy is unclear, changes in smell and taste have been reported in patients who have undergone upper gastrointestinal surgery in clinical settings [7]. Patients often experience a rapid decline in olfactory function after gastrectomy, even before chemotherapy. Rapid malnutrition is likely to cause symptoms of deficiency in each nutrient. For example, zinc is recognized as important not only for taste bud cells but also for olfactory function. We hypothesized that the rapid onset of malnutrition after gastrectomy would also have a significant impact on olfactory function. Olfaction is associated with taste [8] and influences meal satisfaction and nutritional status [9,10]. The impact of changes in olfactory function on postoperative weight loss in gastric cancer patients after gastrectomy is still unknown. Furthermore, it is necessary to clarify the frequency and impact of olfactory changes on postoperative patients.

The aim of this study is to investigate the association between postoperative olfactory changes and postoperative weight loss in gastric cancer patients after gastrectomy. We hypothesize that patients with postoperative olfactory changes will have a higher rate of postoperative weight loss compared to those without postoperative olfactory changes.

## 2. Materials and Methods

### 2.1. Study Design

Patients who underwent radical gastrectomy for gastric cancer at the Cancer Institute Hospital between February 2022 and August 2022 were included. Inclusion criteria were patients aged 20–80 years with a performance status of 0–1 and prior informed consent. Exclusion criteria were (1) patients who had undergone preoperative chemotherapy within one year, (2) patients who had undergone chemotherapy or radiation therapy for any other cancer within one year, (3) patients with dementia, (4) patients receiving zinc supplements, (5) patients with taste disturbance due to other diseases, (6) patients with postoperative complications of Clavien–Dindo classification III or higher, and (7) patients who did not respond to a postoperative questionnaire. The following data were collected prospectively: comorbidities, surgical procedure, surgical approach, clinical stage (cStage), pathological stage (pStage), histopatholgic classification, postoperative complications, use of antibiotics for postoperative complications, body weight preoperatively and 1 month postoperatively, BMI, serum albumin, serum transthyretin, serum zinc, total lymphocyte count (TLC), prognostic nutritional index (PNI), energy intake at 1 month postoperatively, presence of postoperative dumping symptoms, and presence of reflux symptoms at 1 month postoperatively. Energy intake was calculated by a dietitian using the 24 h recall method [11]. The presence or absence of postoperative dumping and reflux symptoms was assessed during nutritional guidance at 1 month postoperatively.

This study was approved by the Ethics Committee of the Cancer Institute Hospital (Approval No.: 2021-GB-093). This study conforms to the “Ethical Guidelines for Medical and Health Sciences Research Involving Human Subjects” of the Ministry of Health, Labour and Welfare of Japan, and complies with the provisions of the Declaration of Helsinki. Patient consent was obtained using an opt-out recruitment method in which all patients were given the opportunity to refuse participation.

### 2.2. Definition of Olfactory and Taste Changes

**Figure 1:**
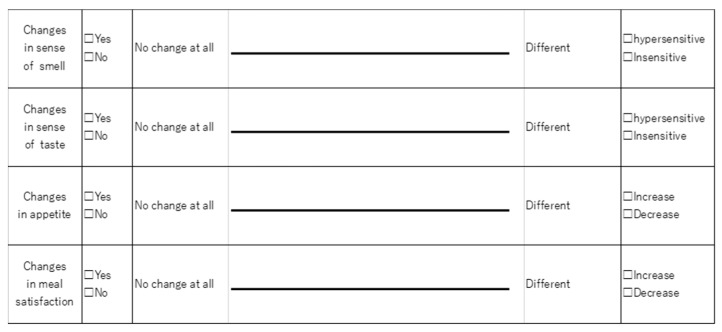
Questionnaire using the Visual Analog Scale.

Olfactory and taste questionnaires were administered preoperatively and one month postoperatively. A dietitian explained the questionnaires before administration and evaluated using the Visual Analog Scale (VAS). The baseline was defined as the condition before the diagnosis of gastric cancer, and the score was obtained by writing a line on the left end of the VAS (0 mm) reading “no change at all” and on the right end (10 mm) reading “change” (Figure 1). The patient then noted the line that applied to the current condition. Patients with a greater VAS score postoperatively compared to preoperatively were defined as having a postoperative olfactory change and a postoperative taste change. Patients who had a change in VAS score were defined as having a positive VAS score for hypersensitivity and a negative VAS score for insensitivity [12].

### 2.3. Outcomes

The primary endpoint was the percentage of postoperative weight loss. Secondary endpoints were the percentage of postoperative taste changes at 1 month postoperatively, the percentage of postoperative appetite loss, the percentage of postoperative loss of dietary satisfaction, postoperative energy intake, and the change in serum transthyretin and serum zinc at 1 month postoperatively. These were compared between groups with and without postoperative olfactory changes.

The rate of weight loss at 1 month postoperatively was calculated as [(preoperative weight − postoperative weight)/preoperative weight × 100]. The cutoff values for postoperative weight loss were defined as the 75th percentile values, and a higher cutoff value was defined as a significant postoperative weight loss.

The same questionnaire was used for appetite and food satisfaction as for olfaction and taste, and patients who reported a greater decrease in VAS values after surgery than before surgery were defined as having an appetite loss and decreased food satisfaction after surgery, respectively.

Postoperative complications were defined as complications of Clavien–Dindo classification II or higher; patients with complications of III or higher were excluded according to study criteria.

### 2.4. Statistical Analysis

All statistical analyses were performed using GraphPad Prism 9^®^ (GraphPad Software Institute Inc., San Diego, CA, USA). Normally distributed continuous variables are presented as mean ± SD and non-normally distributed continuous variables as median (interquartile range: IQR). Wilcoxon signed rank test was used for comparison of two paired groups, and Fisher’s exact test and the Mann–Whitney U test were used for comparison of two unpaired groups. Due to the diversity and substantial variability of cases in histopathological tissues, we conducted Fisher’s exact test to examine the differences between the groups. Correlation coefficients were determined using Spearman’s correlation coefficient. Risk factors for postoperative weight loss were analyzed univariately by logistic regression analysis, and multivariate analysis was performed using factors that were statistically significantly different; *p* < 0.05 was defined as having statistical significance.

## 3. Results

### 3.1. Study Flowchart

**Figure 2:**
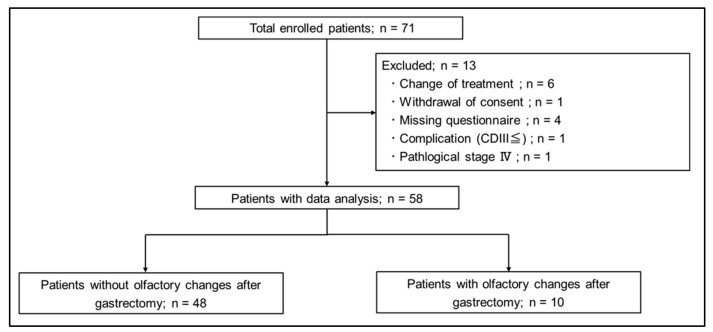
Study flowchart.

Seventy-one patients were prospectively enrolled. A flowchart of the study is shown in Figure 2. A total of 13 patients were excluded: 6 patients (8.5%) due to change of surgical procedure, 1 patient (1.4%) due to withdrawal of consent, 4 patients (5.6%) due to no postoperative questionnaire, 1 patient (1.4%) due to Clavien–Dindo classification III complication, and 1 patient (1.4%) due to pStage IV. Finally, 58 patients were included in the analysis. Of these 58 patients, 10 patients (17.2%) had a postoperative olfactory change and 48 patients (82.8%) had no change.

### 3.2. Comparison of Smell, Taste, Appetite, and Meal Satisfaction before and after Surgery

The percentages of postoperative olfactory and taste changes, appetite loss, and decreased food satisfaction compared to the preoperative period are shown in Table 1. Of the 10 patients with altered olfaction, 9 (15.5%) had hypersensitivity to smell and 1 (1.7%) had a blunted sense of smell. Taste sensitivity was present in 1 patient (1.7%) preoperatively, but 15 patients (25.9%) had taste changes at 1 month postoperatively (*p* < 0.001). Of the 15 patients who experienced taste changes postoperatively, 12 patients (20.7%) were hypersensitive and 3 patients (5.2%) were blunted. Decreased appetite was observed in 11 patients (19.0%) preoperatively, but increased to 20 patients (34.4%) at 1 month postoperatively (*p* = 0.092). Decreased food satisfaction was significantly more common in 25 patients (43.1%) at 1 month postoperatively, compared to 6 patients (10.3%) preoperatively (*p* < 0.001).

### 3.3. Change in Sense of Smell and Taste from Baseline

**Figure 3:**
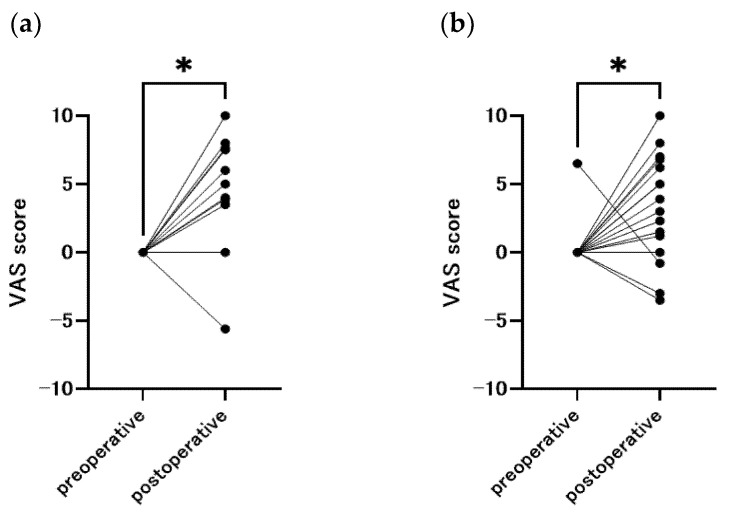
Change in sense of smell and taste from baseline, with a positive Visual Analog Scale indicating hypersensitivity and a negative score indicating insensitivity (n = 58). (**a**) smell (*p* = 0.020), (**b**) taste (*p* = 0.037), * *p* < 0.05.

The changes in VAS scores for olfaction and taste before and after surgery are shown in Figure 3. Compared to the preoperative period, most of the patients had hypersensitive senses of smell and taste after surgery (smell: *p* = 0.020, taste: *p* = 0.037).

### 3.4. Characteristics of Patients with and without Olfactory Changes after Gastrectomy

Table 2 shows the patient backgrounds of the groups with and without olfactory changes. The mean age was 61.2 ± 12.9 years in the group with postoperative olfactory changes and 69.6 ± 7.1 years in the group without postoperative olfactory changes (*p* = 0.075). Patients who received preoperative chemotherapy were excluded from this study. Postoperative chemotherapy was started at least one month after surgery, and this study used an observation period of one month after surgery. Therefore, chemotherapy was not involved in this olfactory change. The rates of total gastrectomy and pylorus-preserving gastrectomy were higher in the group with olfactory changes (*p* = 0.004). We examined whether differentiation type or histological type affected postoperative olfactory changes, but neither histological type nor differentiation type was associated with olfactory changes. Nutrition related markers such as serum albumin, transferrin, zinc, TLC, and PNI were also not associated with the development of postoperative olfactory changes. 

### 3.5. Olfactory Changes and Postoperative Nutrition-Related Indicators

**Figure 4:**
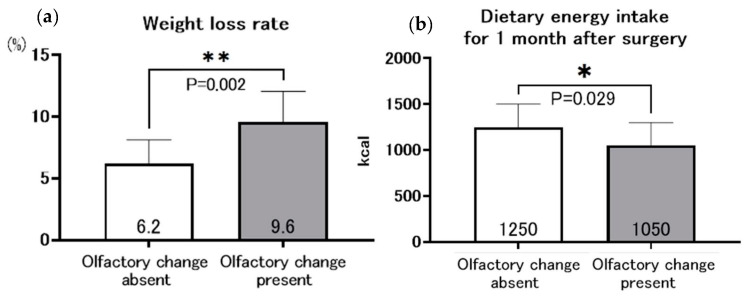
Relationship between olfactory change and weight loss rate (**a**), and dietary energy intake at 1 month after gastrectomy (**b**), * *p* < 0.05, ** *p* < 0.005.

A comparison of the rate of weight loss and oral energy intake at 1 month postoperatively in the group with and without olfactory changes is shown in Figure 4. The rate of weight loss at 1 month postoperatively was significantly greater in the group with olfactory change than in the group without olfactory change (9.6%, 6.2%, respectively; *p* = 0.002). The energy intake was significantly lower in the group with altered olfactory change than without olfactory change (1050 kcal versus 1250 kcal, respectively; *p* = 0.029).

A summary of the comparison of nutrition-related indices is shown in Table 3. The postoperative change in serum transthyretin was significantly lower in the group with olfactory change than in the group without olfactory change (−32.9% vs. −25.9%, respectively; *p* = 0.028). In contrast, the postoperative change in serum zinc levels was higher in the olfactory-change group than in the no-olfactory-change group (4.2% vs. −5.5%, respectively; *p* = 0.038). The group with olfactory changes had a significantly higher percentage of taste changes (*p* < 0.001), decreased appetite (*p* = 0.012), and a lower level of food satisfaction (*p* = 0.014). There were no significant differences in the rates of postoperative dumping symptoms and postoperative reflux symptoms between the two groups.

### 3.6. Relationship between Taste Changes and Olfactory Changes

**Figure 5:**
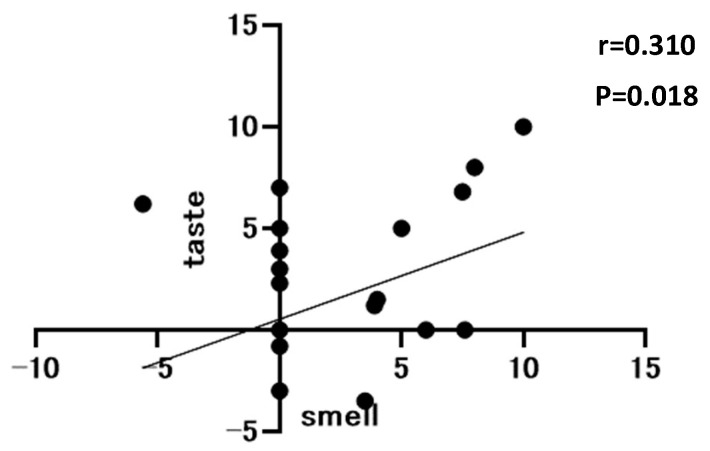
Correlation between olfactory and taste Visual Analog Scale at 1 month postoperatively, n = 58.

The correlation between postoperative olfactory and taste changes is shown in Figure 5. A weak correlation was observed between olfactory and taste changes (r = 0.310, *p* = 0.018).

### 3.7. Risk Factors for Significant Postoperative Weight Loss Rate

Since the 75th percentile of postoperative weight loss in this study was 8.7%, we defined significant weight loss as 8.7% or greater. The results of the analysis of risk factors related to the significant postoperative weight loss are shown in Table 4. Univariate analysis using logistic regression analysis showed statistically significant differences for pStage II or III (*p* = 0.042), total gastrectomy (*p* = 0.045), olfactory changes (*p* = 0.001), and taste changes (*p* = 0.014). Multivariate analysis including these factors identified the olfactory change as an independent risk factor for significant postoperative weight loss (odds ratio: 7.64, 95% confidence interval: 1.09–71.85, *p* = 0.048).

Table 5 shows the results of the analysis of risk factors associated with significant postoperative weight loss with the additional factor of olfactory and taste changes. Univariate analysis using logistic regression analysis showed statistically significant differences in olfactory and taste changes (*p* = 0.002). Multivariate analysis including pStage II or III, total gastrectomy, and olfactory and taste changes identified olfactory and taste changes as an independent risk factor for significant postoperative weight loss (odds ratio: 13.7, 95% confidence interval: 2.27–116.6, *p* = 0.007).

## 4. Discussion

In this study, we investigated the association between postoperative olfactory changes and postoperative weight loss in patients with gastric cancer after gastrectomy. The results showed that olfactory changes occurred in 17.2% of patients after gastrectomy for gastric cancer, and the rate of postoperative weight loss was significantly greater in the group with olfactory changes than in the group without olfactory changes. Multivariate analysis revealed that the olfactory change was an independent risk factor for postoperative weight loss. Correspondingly, patients with olfactory changes had lower dietary energy intake at one month postoperatively. There are several points to discuss in this study. First, patients with olfactory changes had greater weight loss. Second, patients with olfactory changes had lower appetite and postoperative nutrition-related indices but higher serum zinc levels. Third, patients with olfactory changes often had concurrent taste changes. Fourth, the mechanism by which olfactory changes occur postoperatively is unclear.

First, olfactory changes after gastrectomy for gastric cancer were an independent risk factor for weight loss in the first postoperative month. A previous large cohort study showed that olfactory changes influence weight loss in older adults [13]. Hubert et al. [14] reported that patients with olfactory change after bariatric surgery had a higher rate of weight loss, and Lopes et al. [15] reported that patients with olfactory change after bariatric surgery continued their weight loss postoperatively. These results suggest that olfactory changes triggered by gastrectomy are associated with weight loss, which is consistent with the results of this study. The influence of the area of gastric resection on the presence or absence of olfactory changes was also compared. Patients who underwent total gastrectomy and pylorus-preserving gastrectomy were more likely to have olfactory changes. This suggests that pyloric preservation may be important for olfaction. In our previous animal studies, we found that gastric taste receptors are particularly abundant in the fenestra and that patients who underwent fenestral preservation had a preserved sense of taste (unpublished data). In the present study, taste disturbance correlated with olfactory changes, suggesting the importance of the fenestra in the maintenance of olfactory changes as well. However, the relationship between the fumarole and olfaction requires further study.

Second, patients with olfactory changes had lower appetite, postoperative nutrition-related indices, and dietary satisfaction, while serum zinc levels increased. Patients with olfactory change had lower oral energy intake in the first postoperative month and a greater rate of decrease in transthyretin, which is a measure of short-term nutritional status. The sense of smell has previously been reported to influence appetite [16]. In addition, weight loss is associated with decreased serum transthyretin [17]. Therefore, patients with olfactory changes may have more weight loss and lower transferrin due to poor oral intake associated with decreased appetite. On the other hand, patients with olfactory changes had elevated zinc levels. One possible reason for this is that our hospital provides pre-discharge dietary counseling to patients with decreased appetite to consume small amounts of high-energy nutritional supplements. The intake of dietary supplements may have affected zinc concentrations, but the causal relationship is unknown because the foods consumed were not investigated. Patients taking zinc supplements were excluded from this study. One limitation of this study is that we did not examine the intake of other supplements, including vitamins and minerals. Further studies are needed to clarify the relationship between olfactory changes and zinc after gastrectomy.

Third, many patients with postoperative olfactory changes had taste changes. Using Pearson’s correlation coefficient, there was a weak correlation between postoperative olfactory changes and taste changes. A previous report has shown that taste and smell are well related [8]. The brain uses both olfactory and gustatory information to judge the flavor of a meal [18]. In the past, there have been reports of loss of both taste and smell in patients after upper gastrointestinal tract surgery [7]. There are also many reports of both olfactory and taste changes occurring in bariatric surgery for obesity [19,20,21,22]. These results suggest that olfactory changes after gastrectomy may be associated with taste changes in patients with gastric cancer. However, the present study did not elucidate whether taste or olfactory changes occurred first or by a different mechanism. Therefore, further research on the mechanisms by which they occur is needed.

The mechanism by which olfactory changes occur after gastrectomy is unknown. One possible mechanism is the involvement of the vagus nerve. Previous studies have shown that vagus nerve stimulation affects olfaction [23]. In gastrectomy, vagotomy with lymph node dissection may be associated with olfactory changes. The second reason is the involvement of hormonal changes. In patients who have undergone bariatric surgery, leptin concentration and olfactory perception are negatively correlated [24]. Patients with altered olfactory perception in this study also lost significantly more weight, and fat cells were also likely to have decreased, suggesting that leptin secretion may have changed. The third reason is the involvement of gastrointestinal symptoms after gastrectomy. A previous study showed that gastric insufficiency paralysis and the severity of gastroesophageal reflux symptoms correlated with olfactory changes [25]. Although we found no difference in this study, we should consider whether post-gastrectomy symptoms, including reflux symptoms, are involved in patients with olfactory changes. Further research is needed to determine the mechanism of olfactory changes after gastrectomy.

There are advantages and disadvantages to defining olfactory changes by means of a questionnaire based on subjective symptoms. One advantage is that the questionnaire is easy to administer and can be applied immediately in daily clinical practice. Based on the results of this study, weight loss may be predicted simply by determining whether the patient has subjective olfactory symptoms. One disadvantage is that it is unclear whether the sense of smell really changes because objective indicators have not been developed. There are two ways to evaluate olfactory disorders: subjective tests, such as reference olfaction tests, which obtain results based on the subject’s subjective response to a given odor, and objective tests, such as olfactory epithelial and brain response measurement, which detect and indicate biological changes when an odor stimulus is applied [26]. However, olfactory changes have sometimes been reported to cause a discrepancy between subjective symptoms and objective assessment [27]. Even if objective tests show no olfactory changes, the subjective symptoms of patients may indicate anorexia and deterioration of nutritional status, as in this study. Hence, subjective symptoms that are easy to use in daily clinical practice are important.

There are several limitations in this study. First, the mechanism of olfactory change is unknown. Further basic research is needed to determine this mechanism. Second, the sample size was small and the 95% confidence interval of the multivariate analysis was large. Further studies with larger sample sizes are needed. Third, the method used to evaluate olfactory changes was a questionnaire based on subjective symptoms, which did not evaluate objectively. In the future, it is necessary to objectively investigate the olfactory changes using an olfactory test and to examine the relationship between olfactory changes and postoperative weight loss. Despite the limitations of this study, we are the first to show that patients undergoing gastrectomy for gastric cancer have greater postoperative weight loss due to olfactory changes. The impact of this study on daily clinical practice is significant because olfactory changes are not even assessed without attention on the part of healthcare providers. Future studies are needed to determine whether nutritional intervention can reduce postoperative weight loss in patients with olfactory changes.

## 5. Conclusions

Patients with gastrectomy for gastric cancer presented increased olfactory change postoperatively, and olfactory change was an independent risk factor for postoperative weight loss. In addition, patients with olfactory changes showed a greater decrease in nutrition-related indices, such as energy intake and serum transthyretin, as well as decreased dietary satisfaction. Further studies are needed to determine the mechanism of olfactory dysfunction after gastrectomy and to determine whether nutritional intervention in patients with olfactory changes can reduce postoperative weight loss.

## Figures and Tables

**Table 1:** Comparison of smell, taste, appetite, and meal satisfaction before and after surgery.

	Pre-Operation (n = 58)	Post Operation (n = 58)	*p*-Value
Smell alteration	0 (0%)	10 (17.2%)	0.001
Hypersensitivity	0 (0%)	9 (15.5%)	
Insensitivity	0 (0%)	1 (3.1%)	
Taste alteration	1 (1.7%)	15 (25.9%)	<0.001
Hypersensitivity	1 (1.7%)	12 (20.7%)	
Insensitivity	0 (0%)	3 (5.2%)	
Appetite loss	11 (19.0%)	20 (34.4%)	0.092
Decreased meal satisfaction	6 (10.3%)	25 (43.1%)	<0.001

**Table 2:** Characteristics of patients with and without olfactory changes after gastrectomy.

		All (n = 58)	Olfactory Change Absent (n = 48)	Olfactory Change Present (n = 10)	*p* Value
Age (years), mean ± SD		62.6 ± 12.6	61.2 ± 12.9	69.6 ± 7.1	0.075
BMI (kg/m^2^), mean ± SD		23.8 ± 3.8	23.6 ± 3.0	24.5 ± 6.8	0.546
Sex	Male	37 (63.8%)	30 (62.5%)	7 (70.0%)	0.946
	Female	21 (36.2%)	18 (37.5%)	3 (30.0%)	
Performance status	0	55 (94.8%)	45 (93.6%)	10 (100%)	0.999
	1	1 (5.2%)	3 (6.2%)	0 (0%)	
Comobidities					
Diabetes mellitus		9 (15.5%)	8 (16.7%)	1 (10.0%)	0.999
Hypertention		16 (27.6%)	14 (29.2%)	2 (20.0%)	0.541
Hyperlipidemia		4 (6.9%)	3 (6.2%)	1 (10.0%)	0.900
Chronic kidney disease		1 (1.7%)	1 (2.1%)	0 (0%)	0.999
Surgical procedure					
Total gastrectomy		10 (17.2%)	6 (12.5%)	4 (40.0%)	0.004
Proximal gastrectomy		12 (20.7%)	11 (22.9%)	1 (10.0%)	
Distal gastrectomy		27 (46.6%)	26 (54.2%)	1 (10.0%)	
Pylorus-preserving gastrectomy		9 (15.5%)	5 (10.4%)	4 (40.0%)	
Surgical approach					
Laparoscopy		38 (65.5%)	29 (60.4%)	9 (90.0%)	0.141
Robot-assisted		20 (34.5%)	19 (39.6%)	1 (10.0%)	
cStage	I	49 (84.5%)	42 (87.5%)	7 (70.0%)	0.177
	II, III	9 (15.5%)	6 (12.5%)	3 (30.0%)	
pStage	I	45 (77.6%)	39 (81.2%)	6 (60.0%)	0.208
	II, III	13 (22.4%)	9 (18.8%)	4 (40.0%)	
Histopathological classification					
Tubular adenocarcinoma		27 (46.5%)	23 (47.9%)	4 (40.0%)	0.710
Papillary adenocarcinoma		3 (5.2%)	2 (4.2%)	1 (10.0%)	
Poorly differentiated adenocarcinoma		20 (34.5%)	16 (33.3%)	4 (40.0%)	
Signet-ring cell carcinoma		7 (12.1%)	7 (14.6%)	0 (0%)	
Mucinous adenocarcinoma		1 (1.7%)	0 (0%)	1 (10%)	
Postoperative complications		3 (5.2%)	2 (4.2%)	1 (10.0%)	0.074
Antibiotic use present		2 (3.4%)	1 (2.1%)	1 (10.0%)	0.318
Preoperative laboratory data					
Serum albumin (g/dL), median (IQR)		4.3 (4.1, 4.4)	4.3 (4.2, 4.4)	4.3 (4.1, 4.4)	0.709
Serum transthyretin (mg/dL), median (IQR)		29.8 (25.1, 32.9)	29.8 (25.4, 32.9)	30.0 (21.8, 32.7)	0.750
Serum zinc (mg/dL), median (IQR)		80.0 (73.0, 89.0)	80.0 (73.0, 89.0)	80.0 (74.0, 84.9)	0.789
Total lymphocyte count (μL)		1575 (1265, 1968)	1580 (1355, 1990)	1410 (903, 1840)	0.243
Preoperative PNI		50.9 (49.0, 53.0)	50.9 (49.1, 53.2)	51.8 (45.9, 52.7)	0.379

SD: standard deviation, BMI: body mass index, IQR: interquartile range, PNI: prognostic nutritional index

**Table 3:** Comparison of nutrition-related outcomes in patients with or without olfactory change.

	Olfactory Change Absent (n = 48)	Olfactory Change Present (n = 10)	*p*-Value
Weight loss rate at one month (%)	6.2 (3.9, 8.1)	9.6 (6.6, 12.0)	0.002
Change rate of transthyretin at one month (%)	−25.9 (−31.8, −18.3)	−32.9 (−33.9, −29.2)	0.028
Change rate of zinc at one month (%)	−5.5 (−16.2, 1.2)	4.2 (−2.5, 21.7)	0.038
Postoperative status at one month			
Dietary intake (kcal), median (IQR)	1250 (1100, 1500)	1050 (748, 1250)	0.029
Dumping syndrome	10 (20.8%)	2 (20.0%)	0.991
Gastroesophageal reflux disease	14 (29.2%)	6 (60.0%)	0.078
Taste alteration	6 (12.5%)	8 (80.0%)	<0.001
Appetite loss	20 (41.7%)	9 (90.0%)	0.012
Decreased meal satisfaction	22 (45.8%)	9 (90.0%)	0.014

IQR: interquartile range.

**Table 4:** Results of analysis of risk factors for postoperative body weight loss after gastrectomy.

		Univariate Analysis		Multivariate Analysis	
Variables	Category	OR (95% CI)	*p*-Value	OR (95% CI)	*p*-Value
Age (years)	<75	1			
	≥75	1.06 (0.21–4.32)	0.938		
Sex	Female	1			
	Male	2.54 (0.68–12.4)	0.196		
Preoperative BMI (kg/m)^2^	<22	1			
	≥22	0.94 (0.29–4.37)	0.944		
pStage	I	1		1	
	II/III	3.96 (1.04–15.44)	0.042	3.35 (0.58–18.95)	0.163
Postoperative complications	Absent	1			
	Present	7.17 (0.64–162.1)	0.120		
Olfactory change	Absent	1		1	
	Present	13.7 (3.06–76.69)	0.001	7.64 (1.09–71.85)	0.048
Taste change	Absent	1		1	
	Present	5.3 (1.42–20.74)	0.014	1.89 (0.20–13.94)	0.545
Surgical procedure	DG/PG/PPG	1		1	
	TG	4.33 (1.02–18.98)	0.045	1.74 (0.23–11.29)	0.566

OR: odds ratio, CI: confidential interval, BMI: body mass index, DG: distal gastrectomy, PG: proximal gastrectomy, PPG: pylorus-preserving gastrectomy, TG: total gastrectomy.

**Table 5:** Additional analysis of risk factors for postoperative body weight loss after gastrectomy.

		Univariate Analysis		Multivariate Analysis	
Variables	Category	OR (95% CI)	*p*-Value	OR (95% CI)	*p*-Value
Age (years)	<75	1			
	≥75	1.06 (0.21–4.32)	0.938		
Sex	Female	1			
	Male	2.54 (0.68–12.4)	0.196		
Preoperative BMI (kg/m)^2^	<22	1			
	≥22	0.94 (0.29–4.37)	0.944		
pStage	I	1		1	
	II/III	3.96 (1.04–15.44)	0.042	3.47 (0.69–17.01)	0.120
Postoperative complications	Absent	1			
	Present	7.17 (0.64–162.1)	0.120		
Olfactory change	Absent	1			
	Present	13.7 (3.06–76.69)	0.001		
Taste change	Absent	1			
	Present	5.3 (1.42–20.74)	0.014		
Olfactory and taste changes	Absent	1		1	
	Present	15.8 (3.04–122.0)	0.002	13.7 (2.27–116.6)	0.007
Surgical procedure	DG/PG/PPG	1		1	
	TG	4.33 (1.02–18.98)	0.045	1.66 (0.22–9.87)	0.591

OR: odds ratio, CI: confidential interval, BMI: body mass index, DG: distal gastrectomy, PG: proximal gastrectomy, PPG: pylorus-preserving gastrectomy, TG: total gastrectomy.

## Data Availability

The datasets generated and/or analyzed during the current study are available upon reasonable request from the corresponding author.

## Abbreviations

QOL: Quality of life, BMI: body mass index, cStage: stage, pStage: pathological stage, VAS: Visual Analog Scale, TLC: total lymphocyte count, PNI: prognostic nutritional index.
